# EMBRACING OUR DIVERSITY: ENHANCING THE GEROFIT PROGRAM FOR PATIENT EXPERIENCE

**DOI:** 10.1093/geroni/igac059.1271

**Published:** 2022-12-20

**Authors:** Katherine Hall, Monica Serra

## Abstract

The Veterans Health Administration has long been at the forefront of telehealth development and advances. Gerofit to Home (GTH) is a novel, telehealth-based adjunct to a widely successful Gerofit facility-based program for Veterans ages 65 and older with multi-morbidities and functional limitations. Of note, participants are allowed to remain in the program as long as they wish, all of which enhances social engagement and camaraderie. Now disseminated to nearly 30 VA medical centers across the country, all of our sites transitioned within 6 weeks of the COVID-19 pandemic to group-based virtual classes, aptly called GTH. Because of the rapid and continuously evolving changes in telehealth, we had near daily Veteran feedback on what was working and what was not working. We cast a wide net and used multiple methods to obtain Veteran feedback. These were quite successful and allowed us to make ongoing improvements to our classes. The first presentation describes results from a telephone survey deployed to all patients who transitioned from facility-based Gerofit to GTH. The second presentation describes the development of a Gerofit Veterans Advisory Panel and the perspectives they provided in terms of scheduling, patient-requested content, feasibility and access. The third presentation explores patient satisfaction and retention in GTH participants, with an emphasis on rural vs non-rural settings. The fourth paper details an innovative approach to integrating student learners in to the Gerofit program, and explores the impact of these inter-generational interactions.

